# An Hsp70 Chaperone Is Involved in Meiotic Silencing by Unpaired DNA

**DOI:** 10.3390/epigenomes10010007

**Published:** 2026-01-26

**Authors:** Victor T. Sy, Sterling S. Trawick, Hagen M. Tatarsky, Patrick K. T. Shiu

**Affiliations:** Division of Biological Sciences, University of Missouri, Columbia, MO 65211, USA

**Keywords:** genome defense, heat shock proteins (HSPs), meiotic silencing by unpaired DNA (MSUD), *Neurospora crassa*, RNA interference (RNAi)

## Abstract

In the filamentous fungus *Neurospora crassa*, a gene not having a pairing partner during meiosis is seen as a potential intruder and is targeted by a mechanism called meiotic silencing by unpaired DNA (MSUD). MSUD employs core RNA interference (RNAi) components such as the SMS-2 Argonaute, which uses small interfering RNAs (siRNAs) as guides to seek out mRNAs from unpaired genes for silencing. In *Drosophila melanogaster*, the heat shock protein 70 (Hsp70) chaperone system facilitates the conformational activation of an Argonaute and allows it to load siRNAs. Here, our results demonstrate that an Hsp70 protein in *Neurospora* interacts with SMS-2 and mediates the silencing of unpaired genes.

## 1. Introduction

Transposons and other selfish genetic elements can proliferate and wreak havoc on the host chromosomes [1]. It comes as no surprise that many organisms have preserved surveillance mechanisms to protect their genome integrity. In the filamentous fungus *Neurospora crassa*, one such mechanism is known as meiotic silencing by unpaired DNA (MSUD) [2,3,4]. In MSUD, a gene not aligned with a homologous partner can be recognized during a direct dsDNA–dsDNA (double-stranded DNA) pairing event, presumably before meiotic recombination [5]. This pairing process may involve SAD-6 (chromatin remodeler) and REC8 (meiotic kleisin) [6,7]. An aberrant RNA (aRNA) is made from the unpaired DNA and subsequently exported to the perinuclear region, where a host of silencing-related proteins await [8]. There, in conjunction with the SAD-3 helicase, the SAD-1 RNA-directed RNA polymerase turns the single-stranded aRNA into double strands (dsRNA) [9,10]. The dsRNA is chopped up by the DCL-1 Dicer into small interfering RNAs (siRNAs), which are then loaded onto the SMS-2 Argonaute [11,12,13]. With the help of the QIP exonuclease, the passenger strand of an siRNA duplex is removed, and the guide strand is then used to direct SMS-2 to complementary mRNAs bound by nuclear cap-binding proteins NCBP1/2/3 [14,15,16]. The SAD-2 scaffold protein is responsible for anchoring SAD-1 and others to the perinuclear region [8,17,18].

Heat-shock chaperones are proteins that assist in the correct folding of polypeptides, and they are known to promote the conformational activation of Argonaute [19,20]. In *Drosophila*, the Hsp70 (heat shock protein 70) system pries open an empty Argonaute into an active form, which the Hsp90 system then helps stabilize [21]. Several HSP70-related proteins are found in *Neurospora*, including HSP70-1 [22]. HSP70-1 can protect an unfolded protein against aggregation, demonstrating its chaperoning ability [23]. In this study, we explored whether HSP70-1 plays a role in the silencing of unpaired genes in *Neurospora*.

## 2. Materials and Methods

### 2.1. Fungal Manipulation and Genotypes

The *Neurospora* protocol guide was followed during this investigation (https://www.fgsc.net/Neurospora/NeurosporaProtocolGuide.htm; accessed on 19 October 2025). Genotypes of fungal strains used are listed in Table 1. Information for *hsp70-1* (*NCU09602-t26_1*) and other genetic loci used in this study can be found in FungiDB [24]. Various progenitor strains, including the original *hsp70-1* deletion mutant [25], were obtained from the Fungal Genetics Stock Center (FGSC) [26]. Culturing and crossing media were prepared according to standard protocols [27,28].

### 2.2. Assays for Linear Growth, Sexual Sporulation, and MSUD Suppression

Race tubes were used to measure linear growth rates at room temperature [29]. Quantification of ascospore (sexual spore) production was performed according to Hammond et al. [10]. Assessment of MSUD proficiency was essentially as previously described [30], with crosses housed in 24-well microplates and analysis based on shot ascospores. For the above assays, the *p*-values were calculated using the two-tailed Student’s *t*-test.

### 2.3. Protein Tagging and Transformation

Standard molecular biology procedures were followed throughout the course of this work [31]. Green fluorescent protein (GFP) and mCherry tagging constructs were made using double-joint polymerase chain reaction (DJ-PCR) [6,32] and purified with the QIAquick Gel Extraction Kit (QIAGEN, Germantown, MD, USA). *Neurospora* transformation was facilitated by electroporation of conidia (asexual spores) [33]. Primers used in this study are listed in Supplementary Table S1.

### 2.4. Genotype Screening and Confirmation

Genomic DNA was isolated from conidia [34] or vegetative hyphae (filamentous cells) (QIAGEN DNeasy Plant Mini Kit). For PCR-based genotype screening and confirmation, the GoTaq Green Master Mix (Promega, Madison, WI, USA) or the Expand Long Range dNTPack (Roche Diagnostics, Indianapolis, IN, USA) was used. DNA sequencing service was provided by the University of Missouri (MU) Genomics Technology Core.

### 2.5. Bimolecular Fluorescence Complementation (BiFC) Analysis

In BiFC, the N-terminal half of the yellow fluorescent protein (YFPN) is attached to a protein of interest, while the C-terminal half (YFPC) is attached to a potential interactor of that protein [35,36]. If these two tagged proteins interact, a functional yellow fluorophore will be reconstituted. YFPN and YFPC tagging constructs were created using the method of Hammond et al. [32].

### 2.6. Photography and Microscopy Methods

Z-stack pictures of protoperithecia (female structures) were taken by an M205 FA stereomicroscope equipped with a DFC9000 GT camera (Leica Microsystems, Deerfield, IL, USA). To photograph perithecia (fruiting bodies), an Apple iPhone 5 with a Magnifi photoadapter (Arcturus Labs, Lawrence, KS, USA) and a VanGuard 1231CM microscope (VEE GEE Scientific, Vernon Hills, IL, USA) were employed. Images of asci (spore sacs) were captured by a BX45 microscope equipped with a DP74 camera (Olympus, Center Valley, PA, USA). For fluorescence microscopy, preparation and visualization of asci were conducted according to our established procedures [12,14], with the use of a Leica TCS SP8 system at the MU Advanced Light Microscopy Core.

## 3. Results

### 3.1. HSP70-1 Is Important for Meiotic Silencing

In *Drosophila*, heat shock cognate protein 70-4 (Hsc70-4) is a constitutively expressed Hsp70 protein [37,38]. The formation of an active RNA-induced silencing complex (RISC) requires this chaperone, which facilitates the opening of the Ago2 Argonaute for siRNA loading [21,39,40]. Since MSUD also utilizes an Argonaute, we asked whether HSP70-1, the closest homolog of Hsc70-4 in *Neurospora*, is important for silencing.

**Figure 1:**
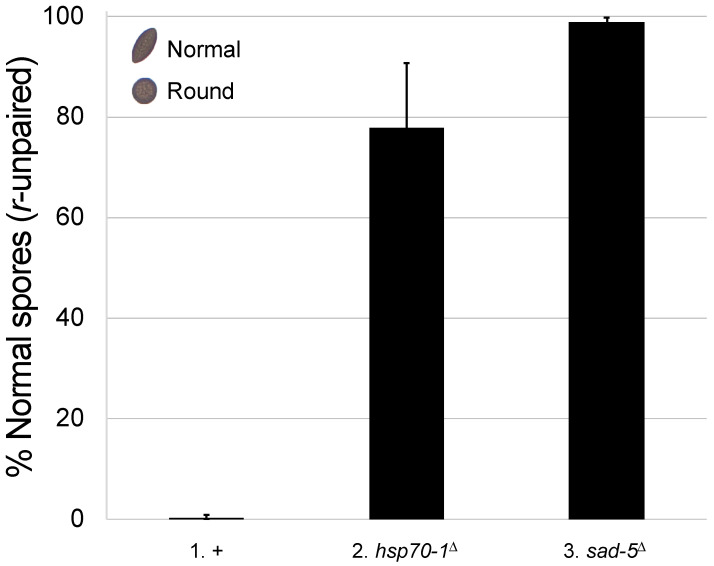
HSP70-1 is important for MSUD. In a normal cross, *Neurospora* produces American football-shaped spores. In a *round spore* (*r*^+^)-unpaired cross (i.e., *r*^+^ × *r*^∆^), *r*^+^ is silenced, and predominantly round spores are produced (i.e., 0.38% football; cross 1). In an *hsp70-1*-null background, the silencing of an unpaired *r*^+^ gene becomes deficient, and significantly more normal spores are produced (i.e., 77.8% football, cross 2; *p* < 0.001). Suppression of silencing is nearly complete (i.e., 98.9% football, cross 3) when the cross is lacking SAD-5 (a protein required for siRNA production) [41]. An error bar indicates the standard deviation among 24 replicates. +, wild type at pertinent loci. Crosses: (1) F9-37 × P3-08. (2) F9-18 × P27-55. (3) F5-36 × P17-70.

In a normal *Neurospora* cross, ascospores are of American football shape. However, if the *round spore* gene is unpaired (i.e., *r*^+^ × *r*^∆^), it will be subject to meiotic silencing, and the resulting progeny will become round [3]. This silencing effect can be alleviated if an MSUD protein is missing. As shown in Figure 1 (cross 2), when HSP70-1 is absent, the silencing of an unpaired *r*^+^ gene is greatly reduced, with the majority (77.8%) of the progeny appearing normal. This suggests that HSP70-1 plays a pivotal role in MSUD.

### 3.2. Mutation in hsp70-1 Affects Vegetative Growth

**Figure 2:**
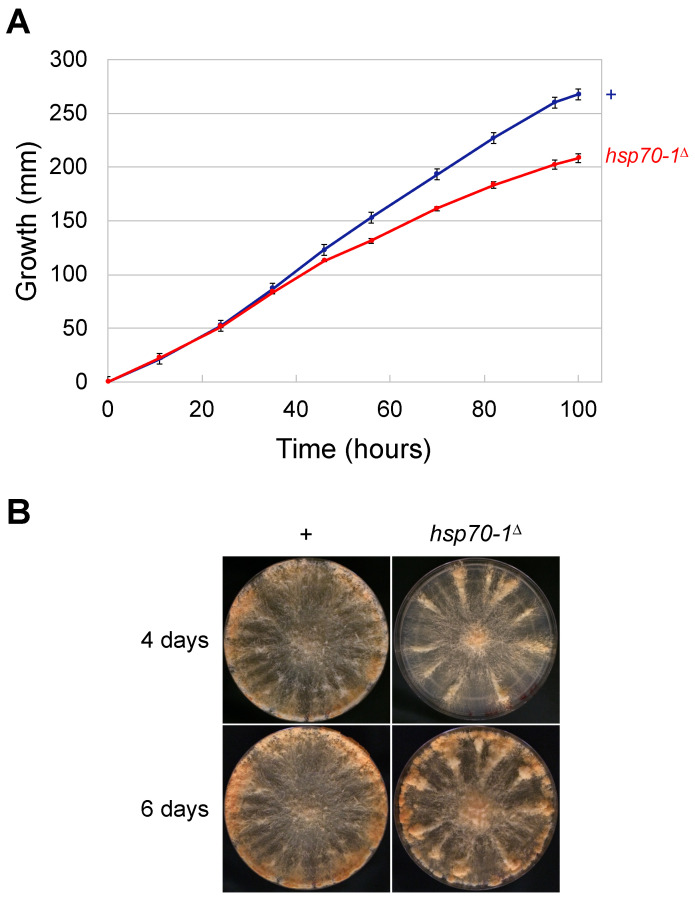
HSP70-1 is involved in the asexual cycle. (**A**) An *hsp70-1*^Δ^ mutant exhibits significantly reduced linear growth when compared to a wild-type strain (208 versus 268 mm at the 100 h mark; *p* < 0.001). The slower growth of the mutant becomes more obvious after around 36 h. (**B**) Conidiation at the perimeter of an agar plate appears proficient (albeit with a small delay) in an *hsp70-1*^Δ^ mutant. An error bar indicates the standard deviation among three replicates. Strains: P3-08 and P27-55.

While meiotic silencing is a sexual phenomenon, certain MSUD mutations notably affect somatic growth. For example, *car-1*^∆^ and *cgh-1*^∆^ mutants are slow growers, with the latter also defective in conidiation pattern [42]. In a race-tube assay, an *hsp70-1*^∆^ mutant achieves only 78% of the linear hyphal growth of a wild-type strain (Figure 2A). When this mutant is grown on an agar plate, conidiation along the edge of the medium appears proficient, albeit slightly delayed (Figure 2B). A previous study on the expression profile of HSP70-1 suggests that it could play a role in conidial formation and germination [43]. In addition, it has been shown that *hsp70-1* mutants have various vegetative defects, e.g., reduced branching and swollen conidia [44]. These past and present findings indicate that HSP70-1 contributes to the normal functioning of the asexual cycle.

### 3.3. HSP70-1 Is Crucial for Sexual Development

**Figure 3:**
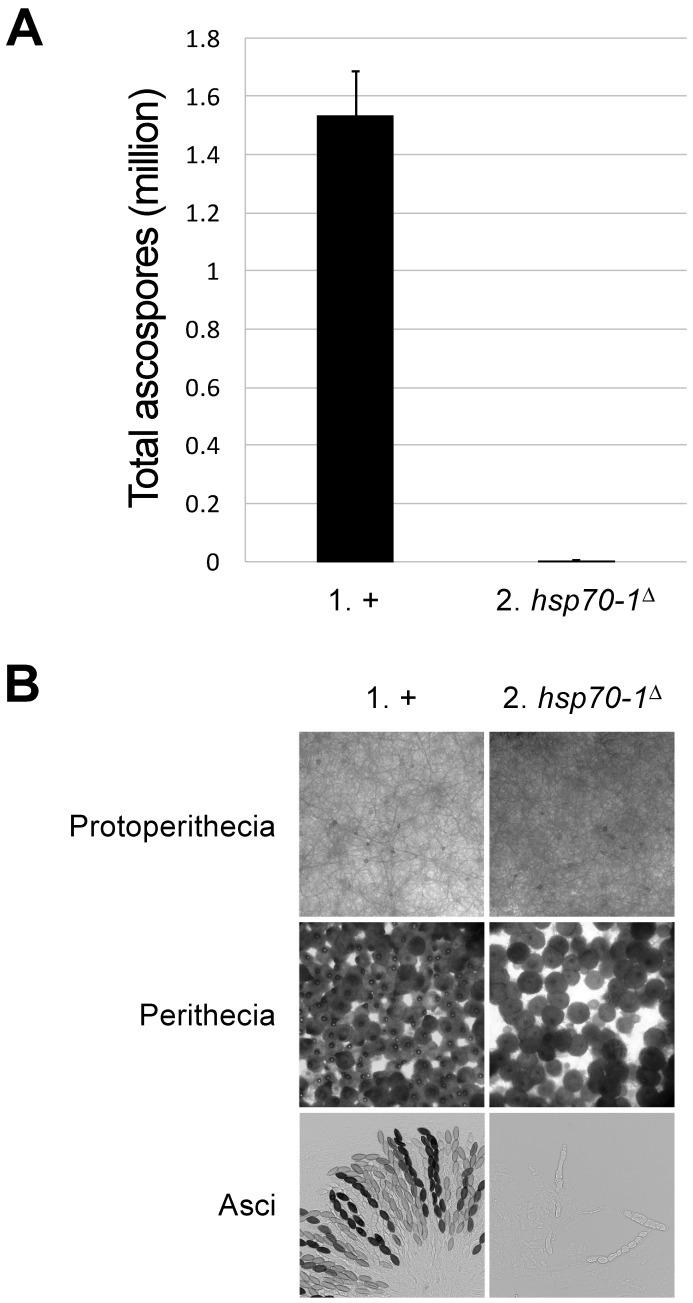
*hsp70-1*-null crosses exhibit severe sexual defects. (**A**) Deletion of *hsp70-1* in both parents leads to a significant decrease in ascospore production (1.53 million versus 3615; *p* < 0.001). (**B**) While protoperithecial development appears proficient in an *hsp70-1*^Δ^ strain, the majority of the mutant perithecia are less melanized and have underdeveloped beaks. Rampant ascus abortions can be seen in these perithecia. An error bar indicates the standard deviation among three replicates. Crosses: (1) F2-01 × P3-08. (2) F9-13 × P27-55.

Many known components of the MSUD pathway are required for sexual reproduction. For example, a cross lacking *dcl-1* or *qip* produces perithecia that are devoid of any asci [12,14]. A less severe phenotype can be observed in a cross lacking *sad-1*, *sad-2*, or *sad-3*, where asci develop but abort before ascospore formation [3,10,17]. In a cross homozygous for an *hsp70-1* deletion, the ascospore production drops by several hundred times (Figure 3A). The mutant perithecia contain mostly abortive asci, suggesting that HSP70-1 is vital to ascus maturation (Figure 3B).

### 3.4. HSP70-1 Is Enriched in the Perinuclear Region

**Figure 4:**
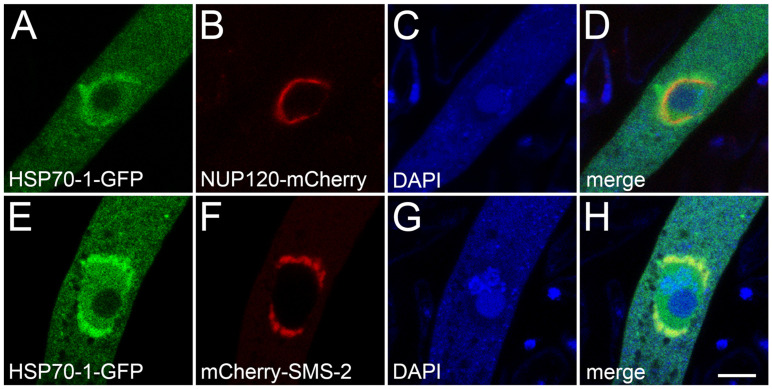
Colocalization of HSP70-1 and SMS-2 in the perinuclear region. (**A**–**D**) HSP70-1 displays a diffused localization throughout the ascus, with a 1.7-fold enrichment surrounding the nuclear envelope (as marked by the NUP120 nucleoporin). (**E**–**H**) HSP70-1 colocalizes with the SMS-2 Argonaute. Micrographs illustrate prophase asci expressing (**A**–**D**) *hsp70-1-gfp* and *nup120-mCherry* (P19-05 × P29-68) and (**E**–**H**) *hsp70-1-gfp* and *mCherry-sms-2* (P29-71 × P29-72). The chromatin was stained with DAPI. Bar, 5 µm.

The perinuclear region, which is immediately outside of the nuclear envelope, is the center of meiotic silencing activity [8,17]. One MSUD protein stationed there is the SMS-2 Argonaute, which uses an siRNA as a guide to look for any homologous mRNAs exported from the nucleus [45]. Since Argonautes are known to have complex formation with Hsp70 proteins [39,46,47], we asked if *Neurospora* HSP70-1 is also found in the perinuclear region. As seen in Figure 4A–D, GFP-tagged HSP70-1 displays a diffused localization throughout the ascus, with an enrichment in the nuclear periphery (i.e., outside of the mCherry-labeled nuclear envelope). Furthermore, when expressed in the same cell, fluorescence-tagged HSP70-1 and SMS-2 colocalize in the perinuclear region, suggesting that they could be in close proximity to each other (Figure 4E–H).

### 3.5. HSP70-1 Interacts with the SMS-2 Argonaute

**Figure 5:**
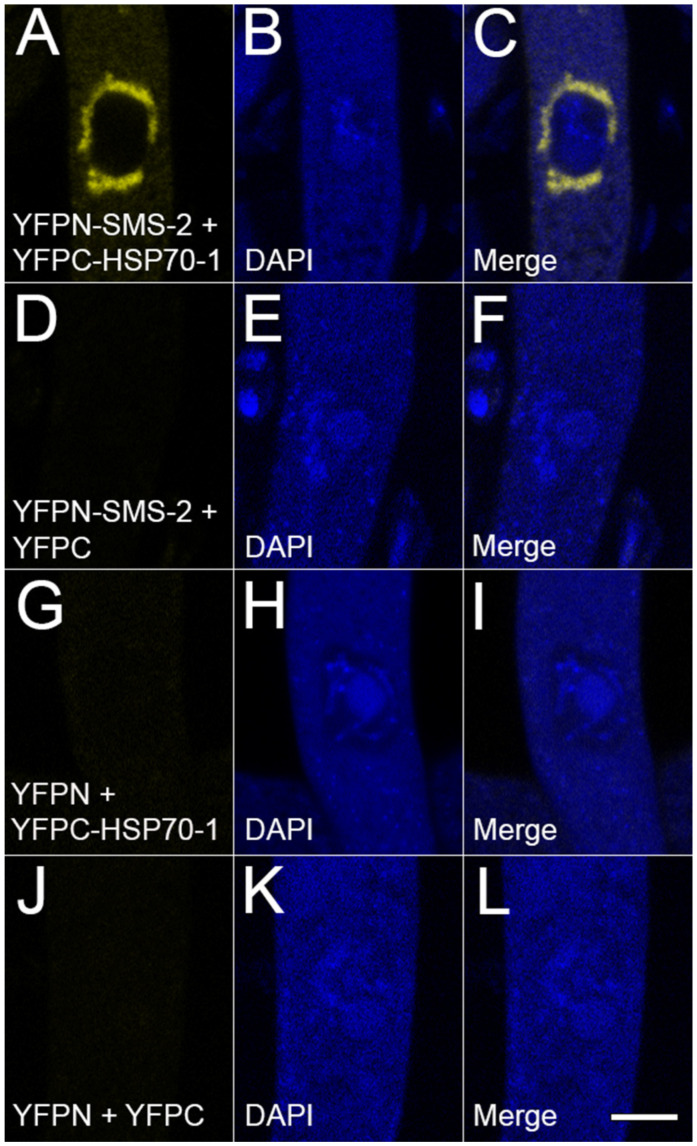
HSP70-1 interacts with SMS-2. In a BiFC analysis, a positive interaction between two proteins reconstitutes the yellow fluorophore. (**A**–**C**) Here, the interaction between HSP70-1 and the SMS-2 Argonaute can be seen in the perinuclear region. (**D**–**L**) Negative controls. Micrographs illustrate a prophase ascus expressing (**A**–**C**) *yfpn-sms-2* and *yfpc-hsp70-1* (P29-57 × P28-31), (**D**–**F**) *yfpn-sms-2* and *yfpc* (P29-57 × P31-03), (**G**–**I**) *yfpn* and *yfpc-hsp70-1* (P31-19 × P28-31), and (**J**–**L**) *yfpn* and *yfpc* (P13-65 × P14-04). The chromatin was stained with DAPI. Bar, 5 µm.

With regard to fungi, although an interaction between Argonaute and heat-shock chaperones has been speculated in *Schizosaccharomyces pombe*, it has yet to be demonstrated [48]. Using a BiFC assay, we tested whether SMS-2 is physically associated with HSP70-1 in *Neurospora*. As shown in Figure 5, SMS-2 indeed has interaction with HSP70-1 in the ascus. This result supports the notion that HSP70-1 could serve as a molecular chaperone for SMS-2.

## 4. Discussion

In animals and plants, the assembly of RISC appears to involve an Hsp70 protein [20]. In fungi, the importance of Hsp70 in silencing had not been established before this study. For example, while Hsp40 and Hsp90 proteins are important for silencing in *S. pombe*, Hsc70-4 homologs (Ssa1 and Ssa2) seem to be dispensable [48]. In the case of *Kluyveromyces polysporus*, purified Argonaute (KpAGO) can autonomously load an siRNA without chaperone proteins or other loading factors [49]. *Naumovozyma castellii* Ago1 also loads an siRNA by itself in vitro, although the loading efficiency can be enhanced by the Xrn1 exonuclease (a non-chaperone factor) [50]. In this work, we have shown that an Hsp70 protein mediates MSUD in *Neurospora*, establishing that it can play a role in fungal silencing.

Crosses lacking HSP70-1 still maintain roughly one-fifth of the MSUD activity. One possibility is that, like *N. castellii* Ago1, SMS-2 can autonomously load an siRNA in some capacity, but it can achieve optimal loading efficiency only when assisted by some helpers (i.e., HSP70-1 and its associated factors in this case). Another possibility is that a homolog of HSP70-1 [22] can partially perform its function in its absence.

While crosses homozygous for some MSUD mutations can produce an appreciable amount of ascospores [6,16,41], this is not the case for an *hsp70-1* deletion. Since the SMS-2 Argonaute is required for ascus formation [51] and that HSP70-1 is its presumed chaperone, the sexual defect of an *hsp70-1*-null cross could be related to an SMS-2 malfunction.

During meiosis, a sequence present in one homologous chromosome but not the other could be a sign of foul play. It is not surprising that some form of meiotic silencing has evolved in various organisms [3,52,53,54,55,56,57]. Here, we have identified HSP70-1 as an additional component of the MSUD pathway. According to the *Drosophila* model, the Hsp70 system could help prime the SMS-2 Argonaute to accommodate an incoming siRNA in *Neurospora*. Since different Argonautes require different accessory proteins for small RNA loading [20], further studies on SMS-2 and its interacting partners could clarify this loading step in meiotic silencing.

## Figures and Tables

**Table 1:** *Neurospora* strains used in this study.

Strain	Genotype
F2-01	*fl A* (FGSC 4317)
F5-36	*fl; sad-5* ^∆^ *::hph a*
F9-13	*rid; hsp70-1* ^∆^ *::hph fl A*
F9-18	*rid r* ^∆^ *::hph; hsp70-1* ^∆^ *::hph fl A*
F9-37	*rid r* ^∆^ *::hph; fl A*
P3-08	Oak Ridge wild type *a* (FGSC 2490)
P13-65	*rid his-3* ^+^ *::yfpn; mus-52* ^∆^ *::bar A*
P14-04	*rid his-3* ^+^ *::yfpc; mus-51* ^Δ^ *::bar a*
P17-70	*r* ^∆^ *::hph; sad-5* ^∆^ *::hph A*
P19-05	*rid nup120-mCherry::hph A*
P27-55	*hsp70-1* ^∆^ *::hph a*
P28-31	*rid; yfpc-hsp70-1::hph; mus-51*^Δ^*::bar A* (heterokaryon with P30-22)
P29-57	*rid; yfpn-sms-2::hph a*
P29-68	*rid nup120-mCherry::hph; hsp70-1-gfp::hph a*
P29-71	*rid;* *mus-51* ^Δ^ *::bar; mCherry-sms-2::nat1 A*
P29-72	*rid; hsp70-1-gfp::hph; mCherry-sms-2::nat1 a*
P30-22	*rid; mus-51* ^Δ^ *::bar A*
P31-03	*rid his-3^+^::yfpc; mus-52* ^∆^ *::bar; mus-51* ^∆^ *::bar A*
P31-19	*rid his-3* ^+^ *::yfpn; mus-52* ^∆^ *::bar a*

## Data Availability

Data and supporting information are contained within the article or Supplementary Materials.

## Supplementary Materials

The following supporting information can be downloaded at: https://www.mdpi.com/article/10.3390/epigenomes10010007/s1. Table S1: Primers for strain construction and confirmation.
