# Supplementary material for: An Hsp70 Chaperone Is Involved in Meiotic Silencing by Unpaired DNA
Source: Epigenomes. 2026 Jan 26;10(1):7. doi: 10.3390/epigenomes10010007 (PMC12921811; doi:10.3390/epigenomes10010007)
Supplement: Supplementary file 1 [file epigenomes-10-00007-s001.zip › epigenomes-3997549-supplementary.pdf]

Table S1. Primers for strain construction and confirmation.

Purpose	Primer	Sequence (5' to 3')
hsp70-1$^{\Delta}$ confirmation	Hsp70 con LFLP	AAACCCAGGTCAGCCAACTTT
	KO con LFRP	ATCCACTTAACGTTACTGAAATCTCCAAC
	NCU09602-2del F	ACGCTAGTGCCTTATCCCAC
	NCU09602-2del R	CCGGCGATGACTTGGATCT
hsp70-1-gfp construction	HSP70-A	AGCGTGCCAAGCGTACTCTCTCTTCT
	HSP70-GFP1	CAGCGCCTGCACCAGCTCCTGCCCCGTCGACCTCCTCGACGGTGGGGC
	HSP70-GFP2	CTCCTTCAATATCAGTTAACAAGGGCCGCGGAGTTAAACGGGTAT
	HSP70-B	GTTACGTCGGAAGGGTTTCT
	HSP70-C	TCCCAGGTCCACGAGAT
	HSP70-D	GCTGACATGGAAGGGAGTAA
mCherry/*yfpn-sms-2* construction	SMS-2-E	GTCCACTTGGTGCCATTCCCACT
	SMS-2-NGFP1	GCAGCCTGAATGGCGAATGGACGCGCGCGGAGGGTGTCAAAACTCACAA
	SMS-2-NGFP2	CAGGAGCGGGTGCGGGTGCTGGAGCGATGTCTGCTCCTGGCTCTCCC
	SMS-2-F	GTGCCATTCTGCTGCTTCCAGTT
	SMS-2-G	CACTTGCCTACCACGCCATGATT
	SMS-2-H	TGCTCAAACCGCCGTAATTGTTG
nup120-mCherry construction	NCU02742-CT-LL	GTACGACTGTGCAACGGCATAGC
	NCU02742-CT-LR	CAGCGCCTGCACCAGCTCCTGCCCCCGACATGAGGATGTCCCCATCGTCACC
	NCU02742-CT-RL	CTCCTTCAATATCAGTTAACAAGGGCATGTTGGGCTGCTTGATTGTCCT
	NCU02742-CT-RR	CTTGGTGCGGTTTGAGGTTGGT
	NCU02742-CT-NL	AGACAGATAGGTGAAGGCGACAAGG
	NCU02742-CT-NR	AAGTGGAGCAAGCGAGCGAAAG
r$^{\Delta}$ confirmation	RSP-988505F	GCCACCTTTCCAACCCAATAATGC
	RSP-993102R	TGCCTTTTCAGCTGGAGACCAAGAC
yfpc-hsp70-1 construction	HSP70-E	GGCGGAGAGAAAGGGACAGATGG
	HSP70-NGFP1	GCAGCCTGAATGGCGAATGGACGCGCGGTTTGCGTGGAGGGAAG
	HSP70-NGFP2	CAGGAGCGGGTGCGGGTGCTGGAGCGATGGCTCCCGCCGTCGGTAT
	HSP70-F	CGTGGACCTGGGACTTGTCGATCTT
	HSP70-G	CTGTTAAGGCTGCGACTGATG
	HSP70-H	CGGGTGATGGAGGTGTAGAA

Primers for DJ-PCR-based fluorescent tagging were designed according to previous reports [16,32].
